# Synthesis of Helical Phenolic Resin Bundles through a Sol-Gel Transcription Method

**DOI:** 10.3390/gels3010009

**Published:** 2017-02-23

**Authors:** Changzhen Shao, Jiangang Li, Hao Chen, Baozong Li, Yi Li, Yonggang Yang

**Affiliations:** Department of Polymer Science and Engineering, College of Chemistry, Chemical Engineering and Materials Science, Soochow University, Suzhou 215123, China; m18862155867@163.com (C.S.); m18896915896-1@163.com (J.L.); chenhaono.1@hotmail.com (H.C.); libaozong@suda.edu.cn (B.L.); ygyang@suda.edu.cn (Y.Y.)

**Keywords:** nanoparticles, polymers, sol-gel preparation, optical activity

## Abstract

Chiral and helical polymers possess special helical structures and optical property, and may find applications in chiral catalysis and optical devices. This work presents the preparation and formation process of helical phenolic resins through a sol-gel transcription method. A pair of bola-type chiral low-molecular-weight gelators (LMWGs) derived from valine are used as templates, while 2,4-dihydroxybenzoic acid and formaldehyde are used as precursors. The electron microscopy images show that the phenolic resins are single-handed helical bundles comprised of helical ultrafine nanofibers. The diffused reflection circular dichroism spectra indicate that the helical phenolic resins exhibit optical activity. A possible formation mechanism is proposed, which shows the co-assembly of the LMWGs and the precursors.

## 1. Introduction

Chiral and helical structures can often be found in nature, for instance, α-helical polypeptides, double helical nucleic acids, spirulina and spiral shells. In the past decades, various methods have been developed for the synthesis of chiral and helical nano-materials for their potential applications in chiral catalysis and separation [1,2,3]. Remarkable progress has been achieved by a sol-gel transcription method using the self-assemblies of chiral low-molecular-weight gelators (LMWGs) as templates [4,5,6,7]. These chiral LMWGs can self-organize into a variety of chiral nanostructures such as coiled ribbons, twisted nanofibers and bundles [8], and lots of chiral and helical, inorganic or organic materials, such as silica and polysilsesquioxane nanotubes, nanoribbons and nanofibers have been successfully synthesized from these templates [9,10,11,12].

Phenolic resins are widely used as industrial materials because of their thermostability, mechanical stability and acid resistance. In recent years, ordered mesoporous phenolic resins have attracted much attention because they can act as a promising carbon source to fabricate ordered mesoporous carbons [13,14,15]. The organic–organic organization of the phenolic resin precursor with the structure-directing agent—generally triblock copolymers—determined the morphology and pore architecture of the mesoporous phenolic resins [13]. As reported, single-handed helical phenolic resin nanotubes have also been prepared using chiral gelators as the templates [16]. The co-assembly of the chiral templates and the phenolic resin precursors determined the chiral structure and optical activity of the nanotubes. Therefore, it is necessary to further investigate the interaction between the chiral templates and the resin precursors.

In this work, helical phenolic resins are prepared using a pair of bola-type chiral LMWG enantiomers derived from valine as templates, 2,4-dihydroxybenzoic acid and formaldehyde as precursors. The morphology of the product is characterized by taking field-emission scanning electron microscopy (FE-SEM) and transmission electron microscopy (TEM) images. Besides, the optical activity is measured by diffused reflection circular dichroism (DRCD) analysis. A possible formation mechanism is proposed according to the FE-SEM images of the reaction mixture taken during the reaction.

## 2. Results and Discussion

**Figure 1:**
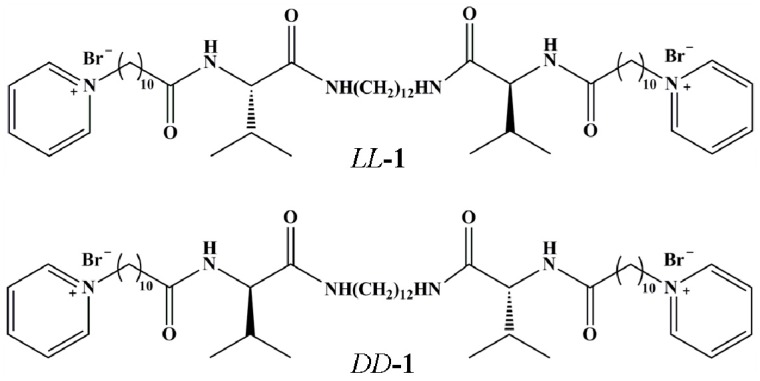
Molecular structures of gelators *LL*-**1** and *DD*-**1**.

**Figure 2:**
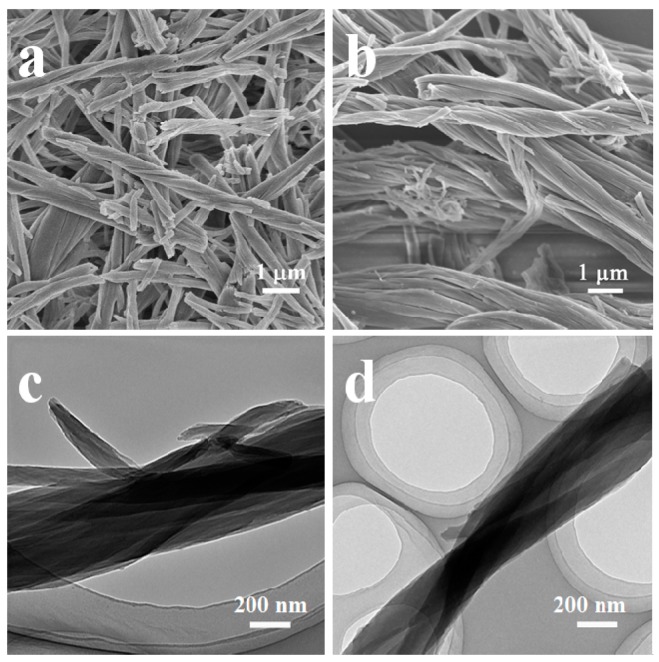
FE-SEM (**a**,**b**); and TEM (**c**,**d**) images of left-handed (**a**,**c**); and right-handed (**b**,**d**) helical phenolic resin bundles.

Bola-type compounds *LL*-**1 (**derived from *L*-valine) and *DD*-**1** (derived from *D*-valine), as shown in Figure 1, can aggregate and form physical gels in deionized water at a concentration of 30 g·L^−1^ at 25 °C [5]. In this work, their self-assemblies are used as the templates to prepared phenolic resins. The FE-SEM and TEM images of the phenolic resin samples are shown in Figure 2. They are single-handed helical bundles comprised of many ultrafine nanofibers. Left-handed helical (Figure 2a) and right-handed helical (Figure 2b) phenolic resins, named as l-HPR and d-HPR, were prepared using *LL***-1** and *DD***-1**, respectively. The diameters of the bundles range from 300 nm to 1 μm while those of the ultrafine nanofibers are uniform at about 40 nm. The lengths of nanofibers can reach tens of microns, although part nanofibers are broken due to the serious stirring. From the TEM images, it is found that the ultrafine nanofibers are helical too (Figure 2c,d). Moreover, there are no clear mesopores identified on the nanofibers. The isothermal nitrogen adsorption–desorption plots for the helical phenolic resins are shown in Figure S1. The samples show type-II isotherms with a capillary-condensation step appearing at relative pressure (*P*/*P*_0_) between 0.8 and 1.0, which originates from voids within and among the bundles. The Barrett–Joyner–Halenda (BJH) pore size distribution plot, determined from the desorption branch, is shown in Figure S1b. The average void width within the bundles is 21 nm. After the d-HPR sample was pyrolyzed at 600 °C for 2.0 h, carbonaceous fibers with diameter of 200–500 nm were obtained (Figure S2). However, no obvious helix could be discerned from Figure S2. It seemed that the surface of helical bundles merged together during carbonization. The possible reason was that the obtained phenolic resin was liner when using 2,4-dihydroxybenzoic acid as the precursor [17].

**Figure 3:**
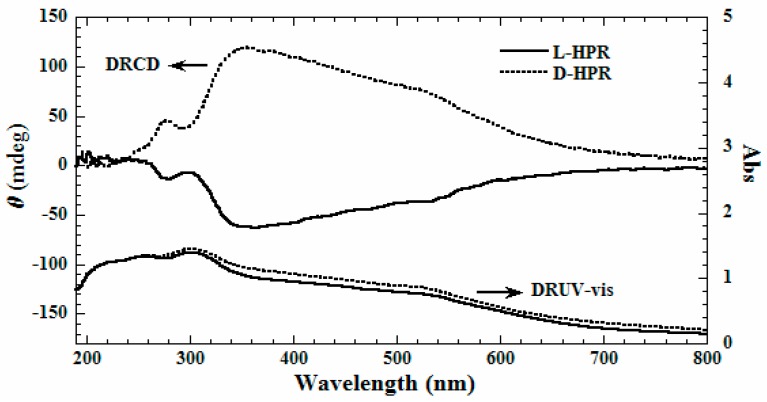
DRUV-vis and DRCD spectra of left-handed and right-handed helical phenolic resin bundles.

The diffused reflection ultraviolet-visible absorption (DRUV-vis) and DRCD spectra of the helical phenolic resins are shown in Figure 3. Broad absorption bands at 200–700 nm are observed on the DRUV-vis spectra. For d-HPR, it shows two positive signals at 355 and 278 nm on the DRCD spectrum. The first positive DRCD signal indicates the right-handed packing of the aromatic rings in the phenolic resins [4]. On the contrary, l**-**HPR exhibits opposite signals at 364 and 280 nm, and the first negative DRCD signal indicates the left-handed packing of the aromatic rings in the phenolic resins. The obtained helical phenolic resins possess optical chirality, which might find potential applications in chiral separation and catalysis.

**Figure 4:**
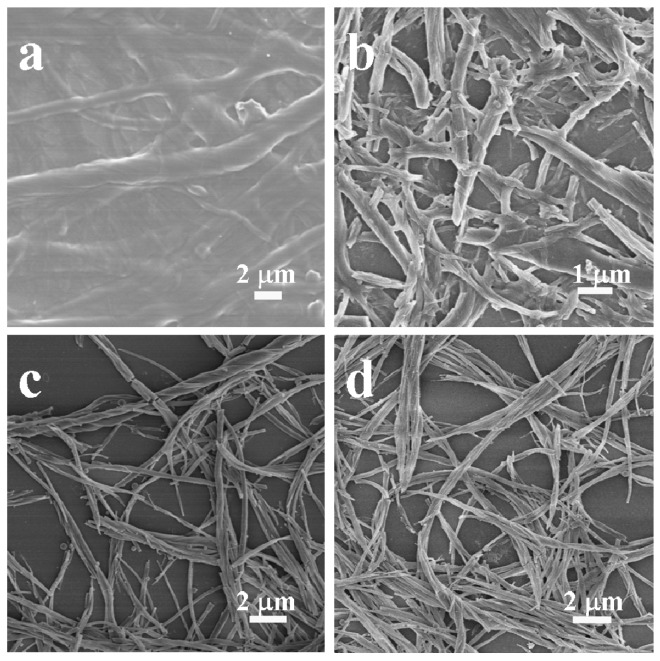
FE-SEM images of the reaction mixture taken at different reaction times: (**a**) before the addition of formaldehyde; (**b**) 5 min after the addition of formaldehyde; (**c**) 10 min after the addition of formaldehyde; (**d**) 30 min after the addition of formaldehyde.

**Figure 5:**
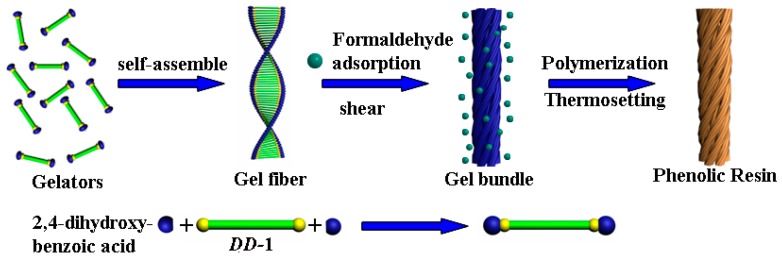
Schematic illustration of the formation of helical phenolic resin bundles.

To investigate the formation process of the helical phenolic resins, the FE-SEM images of the reaction mixture were taken at different reaction times, as shown in Figure 4. Before the addition of formaldehyde, the reaction mixture was a transparent viscous solution. Fibrous aggregates were observed in Figure 4a, and some nanofibers exhibited right-handedness. When the aqueous ammonia solution and formaldehyde were added into the mixture, the solution became cloudy quickly, and brown precipitation appeared after 5 min (Figure 4b). Right-handed helical nanofibers and nanobundles were observed in Figure 4c. The nanofibers became longer with time (Figure 4d). The possible formation mechanism of the helical phenolic resins is illustrated in Figure 5. Firstly, gelator *DD*-**1** combined with 2,4-dihydroxybenzoic acid through electrostatic interaction between the carboxylate radical and pyridinium in the aqueous solution. Meanwhile, the gelator molecules self-assembled through non-covalent interactions to form single-stranded helical fibrous aggregates. The 2,4-dihydroxybenzoic groups were distributed on the two sides of the gel fibers. Under the shear force action, these single-stranded gel fibers twisted together to form helical bundles, as shown in Figure 4a. Secondly, when the aqueous ammonia solution and formaldehyde were added into the reaction mixture, the formaldehyde molecules adsorbed onto the surface of gel fibers and reacted with 2,4-dihydroxybenzoic groups under basic condition. Therefore, the polycondensation reaction was carried out on the outer surface of the helical gel fibers, and a phenolic resin-template composite bundles were soon formed at room temperature, as shown in Figure 4b. At that time, the helical morphology of the composite was basically settled. The following thermosetting reaction at high temperature (90 °C) for a certain time just improved the cross-linking degree of resin. Finally, the organic templates embedded in the phenolic resin nanofibers were removed by ethanol extraction, and the obtained phenolic resins preserved the single-handed helical morphology of the original organic aggregates.

## 3. Conclusions

In summary, single-handed helical phenolic resin bundles were synthesized through a sol-gel transcription approach using a pair of bola-type chiral LMWGs as templates, 2,4-dihydroxybenzoic acid and formaldehyde as precursors. A co-assemble process was proposed for the formation of helical phenolic resin bundles. These helical bundles exhibited optical chirality, and produced carbonaceous fibers after carbonization, which threw light on the synthesis of chiral carbons.

## 4. Materials and Methods

### 4.1. General Methods

TEM images were obtained using an FEI TecnaiG220 (Hillsboro, OR, USA) at 200 kV. FE-SEM was performed using a Hitachi 4800 instrument (Ibaraki prefecture, Japan) at 3.0 kV. DRCD and DRUV-vis spectra were obtained using a JASCO 815 spectrophotometer (Tokyo, Japan). The specific surface area and pore-size distribution were determined by the Brunauer–Emmett–Teller (BET) and BJH methods, using N_2_ adsorption isotherms measured using a Micromeritics Tristar II 3020 instrument (Norcross, GA, USA).

### 4.2. Materials

The compounds *LL*-**1** and *DD*-**1** were synthesized according to the literature [5]. Concentrated ammonium hydroxide aqueous solution (25–28 wt %), formaldehyde (37 wt %), ethanol, 2,4-dihydroxybenzoic acid and methanol were purchased from Sinophram Chemical Reagent Co., Ltd (Shanghai, China).

*Synthetic procedure for helical phenolic resin* (*HPR*): *LL***-1** (or *DD***-1**) (25 mg, 0.025 mmol) and 2,4-dihydroxybenzoic acid (20 mg, 0.13 mmol) were dissolved in 5 mL of distilled water at room temperature to form a viscous solution. Aqueous ammonia solution (40 μL) and formaldehyde (12 μL) were added into the solution. The reaction mixture was stirred at vortex for 1 min, followed by aging at 90 °C for 2 h. The resultant brown precipitate was filtered and dried to yield as-prepared polymer. The organic low-molecular-weight compounds were removed by extracting the as-prepared product with 80 mL of methanol for 24 h. After being further heated in an oven at 100 °C for 24 h for thermosetting, the cross-linked helical phenolic resin was obtained.

## Supplementary Materials

Supplementary data associated with this article can be found in the online version at www.mdpi.com/2310-2861/3/1/9/s1. Figure S1. (a) Nitrogen sorption isotherms and (b) BJH pore size distributions calculated from the desorption branch of l- and d-HPR; Figure S2. FE-SEM image of d-HPR after carbonization.

## References

[1] Dong J., Liu Y., Cui Y. (2014). Chiral porous organic frameworks for asymmetric heterogeneous catalysis and gas chromatographic separation. Chem. Commun..

[2] Li Z., Yao J., Tao Q., Jiang L., Lu T. (2013). Enantioselective recognition and separation of racemic 1-phenylethanol by a pair of 2D chiral coordination polymers. Inorg. Chem..

[3] Kaushik M., Basu K., Benoit C., Cirtiu C., Vali H., Moores A. (2015). Cellulose nanocrystals as chiral inducers: Enantioselective catalysis and transmission electron microscopy 3D characterization. J. Am. Chem. Soc..

[4] Huo H., Li Y., Yuan Y., Lin S., Li B., Wang M., Yang Y. (2014). A single-step approach for fabrication of vancomycin-bonded silica monolith as chiral stationary phase. Chem. Asian J..

[5] Xiao Z., Guo Y., Li B., Li Y. (2016). Preparation of Single-Handed Helical Carbon/Silica and Carbonaceous Nanotubes by Using 4,4′-Biphenylene-Bridged Polybissilsesquioxane. J. Wuhan Univ. Technol. Mater. Sci. Ed..

[6] Moshe H., Levi G., Sharon D., Mastai Y. (2014). Atomic layer deposition of enantioselective thin film of alumina on chiral self-assembled-monolayer. Surf. Sci..

[7] Okazaki Y., Cheng J., Dedovets D., Kemper G., Delville M., Durrieu M., Ihara H., Takafuji M., Pouget E., Oda R. (2014). Chiral Colloids: Homogeneous Suspension of Individualized SiO_2_ Helical and Twisted Nanoribbons. ACS Nano.

[8] Hanabusa K., Yamada M., Kimura M., Shirai H. (1996). Prominent Gelation and Chiral Aggregation of Alkylamides Derived from *trans*-1,2-Diaminocyclohexane. Angew. Chem. Int. Ed..

[9] Huang Z., Yao Y., Han L., Che S. (2014). Control of Chiral Nanostructures by Self-Assembly of Designed Amphiphilic Peptides and Silica Biomineralization. Chem. Eur. J..

[10] Qiu H., Che S. (2008). Formation mechanism of achiral amphiphile-templated helical mesoporous silicas. J. Phys. Chem. B.

[11] Wu X., Crudden C. (2012). Chiral hybrid mesoporous silicas: Assembly of uniform hollow nanospheres and helical nanotubes with tunable diameters. Chem. Mater..

[12] Zhao M., Zhang Q., Tian G., Wei F. (2014). Nanoscale Emerging double helical nanostructures. Nanoscale.

[13] Fang Y., Gu D., Zou Y., Wu Z., Li F., Che R., Deng Y., Tu B., Zhao D. (2010). A Low-Concentration Hydrothermal Synthesis of Biocompatible Ordered Mesoporous Carbon Nanospheres with Tunable and Uniform Size. Angew. Chem. Int. Ed..

[14] Li P., Song Y., Guo Q., Shi J., Liu L. (2011). Tuning the pore size and structure of mesoporous carbons synthesized using an evaporation-induced self-assembly method. Mater. Lett..

[15] Li J., Qi J., Liu C., Zhou L., Song H., Yu C., Shen J., Sun X., Wang L. (2014). A Fabrication of ordered mesoporous carbon hollow fiber membranes via a confined soft templating approach. J. Mater. Chem. A.

[16] Chen H., Tang X., Li Y., Li B., Zhang C., Yang Y. (2015). Preparation of single-handed helical carbonaceous nanotubes using 3-aminophenol-formaldehyde resin. RSC Adv..

[17] Valkama S., Nykänen A., Kosonen H., Ramani R., Tuomisto F., Engelhardt P., Brinke G.T., Ikkala O., Ruokolainen J. (2007). Hierarchical Porosity in Self-Assembled Polymers: Post-Modification of Block Copolymer–Phenolic Resin Complexes by Pyrolysis Allows the Control of Micro- and Mesoporosity. Adv. Funct. Mater..

