# Supplementary material for: Synthesis of Helical Phenolic Resin Bundles through a Sol-Gel Transcription Method
Source: Gels. 2017 Feb 23;3(1):9. doi: 10.3390/gels3010009 (PMC6318678; doi:10.3390/gels3010009)
Supplement: Supplementary file 1 [file gels-03-00009-s001.pdf]

Synthesis of Helical Phenolic Resin Bundles through a Sol-Gel Transcription Method

Changzhen Shao, Jiangang Li, Hao Chen, Baozong Li, Yi Li and Yonggang Yang

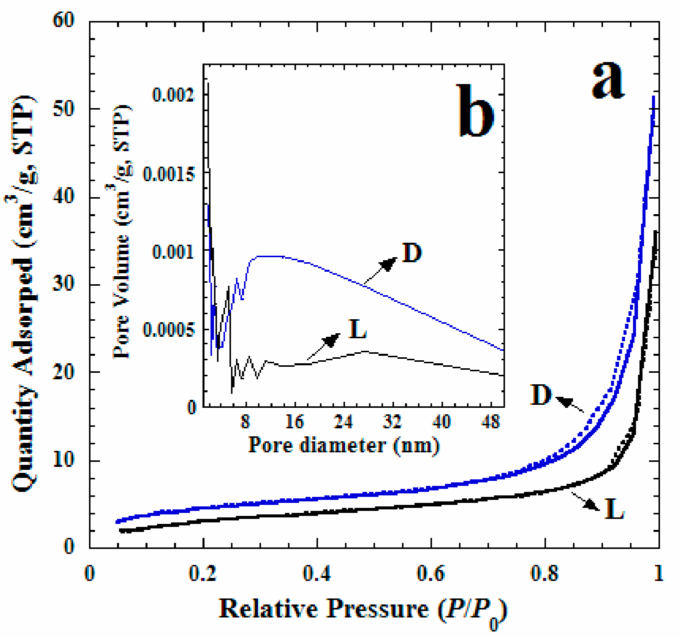

Figure S1. (**a**) Nitrogen sorption isotherms and (**b**) BJH pore size distributions calculated from the desorption branch of L- and D-HPR.

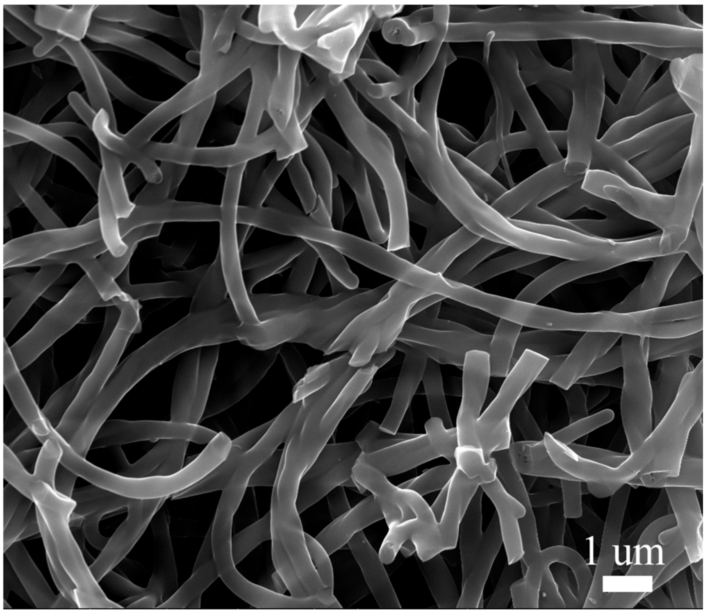

Figure S2. FE-SEM image of D-HPR after carbonization.
